# Stress Spectrum in Pregnancy: Association with Socio-Economic, Educational, and Cultural Factors

**DOI:** 10.19044/esj.2025.v21n39p1

**Published:** 2025-03-15

**Authors:** N. Masiukovichi, E. Nikoleishvili, W. M. Caudle

**Affiliations:** University of Georgia, Tbilisi, Georgia; University of Georgia, Tbilisi, Georgia; Emory University, Atlanta, USA

**Keywords:** Pregnancy, Stress, Socio-Economic Factors, Cultural Aspects, Stress Biomarkers, Saliva Cortisol Levels

## Abstract

**Introduction:**

The multifaceted nature of stress during pregnancy is known to influence maternal health, pregnancy outcomes, and fetal development.

**Objective:**

This study aims to explore the association between stress during pregnancy and socio-economic, educational, and cultural factors in Tbilisi, Georgia.

**Methods:**

A cross-sectional study was conducted among 398 pregnant women in Tbilisi, Georgia, utilizing a pregnancy-specific questionnaire approved by the University of Georgia’s ethics board. Saliva cortisol levels were measured in a focus group of 95 pregnant women who self-reported stress. Statistical analysis was performed using SPSSv.23.0.

**Results:**

Two groups were identified: Group I (n = 172), comprising women who self-reported stress and had elevated cortisol levels (n = 95), and Group II (n = 226), who did not report stress. In Group I, saliva cortisol levels indicated mild stress in 30.5%, moderate stress in 34.7%, and high stress in 34.7%. Occupational stress factors were more prominent in Group I compared to Group II: job-related stress (OR = 1.59, p = 0.02, CI = 95%), overtime work (OR = 3.05, p = 0.001, CI = 95%), and continued work throughout pregnancy (OR = 1.82, p = 0.01, CI = 95%). Environmental stress was more prevalent among women living alone or with a partner (OR = 1.59, p = 0.03, CI = 95%). Relationship stress was significantly lower in Group I (p = 0.01), while psychological stress, such as spontaneous abortion, was higher (OR = 2.19, p = 0.001, CI = 95%). Educational levels did not differ significantly between groups.

**Conclusions:**

Self-reported stress is closely linked to elevated cortisol levels during pregnancy. Findings highlight the importance of addressing psychological, social, and occupational factors in prenatal care, to address the improvement of maternal and fetal health outcomes in the future.

## Introduction

Pregnancy is a time of profound physical and emotional changes for women. While often celebrated as a joyful period, it can also be marked by significant stress and anxiety. The emotional well-being of expectant mothers is influenced by a complex interplay of factors ranging from hormonal changes, fears about childbirth, and the challenges of impending motherhood. Beyond these immediate concerns, broader socio-economic, and cultural influences also play a crucial role in shaping stressful experiences of pregnant women. Psycho-emotional stress during pregnancy, influenced by multiple factors, has become a critical area of research due to its potential impact on both maternal and fetal health (Elysia Poggi Davis, Angela J Narayan, 2021).

Stress during pregnancy is a complex phenomenon that arises from various stressors, including biological changes, personal expectations, and external pressures. However, the social, economic, and cultural contexts in which a woman lives play a pivotal role in shaping the nature and intensity of stress. Socio-economic status, cultural norms, and access to social support are key determinants that influence a pregnant woman’s emotional well-being and her ability to cope with the challenges of pregnancy. Women from lower socio-economic backgrounds often face increased stress, lack of access to quality healthcare, and limited social support. These stressors can exacerbate feelings of anxiety, depression, and uncertainty during pregnancy. In contrast, cultural beliefs and practices surrounding pregnancy can either alleviate or intensify stress. For instance, certain cultural traditions may provide a supportive community network, while others may impose restrictive norms that increase the psychological burden on expectant mothers (WHO. Consequences of stress. 2023).

Prenatal stress-elevated stress levels during pregnancy-have garnered significant attention in the fields of psychology, neuroscience, and public health ( Vivette Glover, 2019; Mulder EJ. et al. 2002). The recognition of the potential long-lasting impact of prenatal stress on the developing fetus and future offspring has prompted extensive research in recent years (Zietlow AL. et al. 2019; Mary E Coussons. 2013).

Prenatal stress encompasses a wide range of stressors, both chronic and acute, that can significantly influence the intrauterine environment. These stressors may include maternal anxiety, depression, exposure to traumatic events, socioeconomic challenges, and environmental factors such as pollution and noise. Prenatal stress is a complex construct, with various contributing factors that can interact and manifest in diverse ways (Kinsella MT, Monk C. 2009). The importance of studying prenatal stress lies in its potential to shape the health and well-being of future generations. Research in this area has expanded our understanding of how early-life experiences can have lasting effects on behavior, cognition, and mental health outcomes. Prenatal stress is not a solitary event but rather a dynamic process that can set in motion a cascade of biological and psychological responses that influence an individual’s trajectory throughout life (Mbiydzenyuy N.E 2022). Animal studies have shown that stress during pregnancy can have long-lasting effects on the neurodevelopment of the offspring (Weinstock M. 2005).

Psychosocial, cultural, and environmental stressors experienced during gestation can be detrimental to pregnancy and maternal and fetal health that span generations (Milad MP, Klock SC. 1998). The majority of human studies show that mild, moderate and severe stress can have negative influences on pregnancy outcome and the behavioral and physiological development of offspring (Lobel M. J Behav. 1994). Several conceptualizations of ‘prenatal stress’ are evident in the human literature, reflecting the diversity of stressors that may be experienced during gestation. The concept of a psychosocial stressor encompasses changes in, for example, personal life, job status, housing, domestic violence and family makeup which require adaptive coping behavior on the part of the affected individual. (Orr ST, James SA. 1992) Prenatal stress can have direct effects on infant health by altering the course of fetal neurobiological development. (Davis EP. 2011) Prenatal stress can indirectly affect infant health and development by increasing the risk of the occurrence of adverse birth outcomes which are, in turn, associated with substantial developmental and health consequences (Dong Y, Yu JL. 2011).

Both the direct and indirect effects of prenatal stress can have long-lasting consequences for both, development, and functioning of offspring across the lifespan (Mary E Coussons-Read. 2013).

A limited number of human studies also document that high-dose synthetic glucocorticoid exposure in pregnancies at risk for preterm delivery has been associated with neurodevelopmental delays, emotional dysregulation, and memory impairments in offspring (French, 1999; Davis et al., 2006).

This study aims to identify the range of stress experienced by pregnant women in Tbilisi, Georgia, and explore how educational, socio-economic, and cultural factors relate to stress levels. A subset of participants (N = 95 out of 398) will have their cortisol levels measured to assess the relationship between chronic stress and saliva cortisol in pregnancy. The research seeks to understand how external factors—such as financial stability, healthcare access, cultural expectations, and societal norms—impact maternal well-being. By revealing these associations, the study aims to provide valuable insights to guide the creation of tailored support systems and interventions designed to reduce stress and enhance maternal health outcomes.

## Methods

A cross-sectional observational study was performed. The questionnaire - as the primary tool of the study, was approved by the ethics committee of the University of Georgia (Research code: UGREC-03–23). Data were analyzed using SPSSv.23.0, with statistical significance assessed by the Chi-squared test (p < 0.05 indicating statistical significance). The research was conducted from April 2023 up to July 2024, in Tbilisi, Georgia.

Target pregnant women were selected from maternity homes and women’s consultations across the city Tbilisi, Georgia. Informed consent was obtained from all participants, and they were assured of the confidentiality of their responses.

Confidence interval: 95%; margin of error: 5%

### Inclusion criteria for the pregnant women included the study:

These criteria were established to create a representative sample of the pregnant population in Tbilisi, while minimizing potential confounding variables that could affect the study’s outcomes.

### Exclusion criteria for the pregnant women included:

The primary data collection tool - the pregnancy questionnaire, was designed to assess the stress environment, psycho-emotional stress, and the related factors included the sections described in the table below (Table 3). The questionnaire comprises 46 questions (with “yes” or “no” questions, questions with multiple answers, and open-ended questions)

### Saliva collection protocol:

Saliva samples were collected from a focus group of 95 pregnant women who self-reported experiencing chronic, continuous stress via questionnaires. Sampling was conducted in the afternoon to measure evening salivary cortisol levels. Evening salivary cortisol is preferred for its diagnostic sensitivity in detecting HPA axis dysfunction and chronic stress-related conditions. Its low baseline levels in the evening provide a more reliable measure for identifying abnormal elevations that might not be apparent in morning samples. To ensure accuracy, participants were instructed to refrain from eating, drinking, or brushing their teeth for at least 30 minutes before collection.

Saliva was collected using sterile tubes provided by Neolab Laboratory (Tbilisi, Georgia), accompanied by clear instructions on the collection procedure. Participants washed their hands with soap and warm water, dried them with a clean towel, and then removed the tube cap to access the swab inside. They were instructed to tip the tube, allowing the swab to drop into their mouths without touching it directly. Each participant held the swab in their mouth for two minutes, moving it gently to ensure full saturation. If movement was difficult, participants placed the swab under their tongues. Chewing the swab was discouraged to maintain sample integrity.

After thoroughly soaking the swab, participants returned it to the tube without direct contact and securely capped the container. Each sample was labeled with the participant’s code, date, and time.

### Laboratory Analysis:

The saliva samples (N = 95), after collection, were stored in sterile tubes, then placed in the mobile refrigerator at a temperature of (+) 4 degrees Celsius (in the dry ice) and transported to the laboratory for analysis. The cortisol concentrations were measured using the enzyme-linked immunosorbent assay (ELISA) technique, a sensitive and accurate method for detecting hormone levels in biological samples. The saliva cortisol was measured by the cortisol ELISA Kit (provided by Enzo Biochem Inc., USA).

The cortisol ELISA kit is a competitive immunoassay for the quantitative determination of cortisol in biological fluids. The kit for the quantitative measurement of cortisol uses a monoclonal antibody to cortisol to bind, in a competitive manner, cortisol in a sample or an alkaline phosphatase molecule that has cortisol covalently attached to it. After a simultaneous incubation at room temperature, the excess reagents are washed away and the substrate is added. After a short incubation time, the enzyme reaction is stopped, and the yellow color generated is read on a microplate reader at 405 nm. The intensity of the bound yellow color is inversely proportional to the concentration of cortisol in either standards or samples.

The collected data were statistically analyzed using SPSSv.23.0 software (IBM Corp., Armonk, NY, USA). Continuous variables are expressed as mean ± standard deviation (SD), checked for normality by Kolmogorov-Smirnov Z-test, and the differences were assessed by analysis of variance (within the groups – paired t-test, between the groups – an independent t-test, and Fisher’s exact test). Categorical variables were compared using the Chi-squared test or Fisher’s exact test. Risk-factors of stress were assessed by the calculation of odds ratio (OR) and 95% confidence intervals (95% CI). P-values of <0.05 were considered statistically significant.

## Results

The findings provide an in-depth understanding of the multi-faceted nature of stress during pregnancy, highlighting the complex interplay between social, economic, environmental, relationship, and psychological factors and maternal well-being.

Participants of the study (N = 398) had been divided into two groups:

### Group 1 (N = 172) –

Pregnant women with stress, confirmed by self-assessment questionnaires and the saliva cortisol concentrations (saliva cortisol randomly measured in N = 95 women out of N = 172).

### Group 2 (N = 226) –

Pregnant women, without stress, confirmed by self-assessment questionnaires.

An independent t-test was applied (t-test =−1.721, p = 0.086) to check the difference of the mean age between Group 1 and Group 2. The age of participants in Group 1 and Group 2 showed no significant difference (Table 4).

Chi-squared test was performed (Chi-squared test = 2.91; p = 0.088) to find out the difference according to the dwelling place between the groups. The results obtained showed that the percentage of participants from the regions of Georgia in Group 1 was not significantly different, compared to the same value of Group 2 (Table 5).

The results of the Chi-squared test (Chi-squared test = 0.66; p = 0.719) showed no statistically significant difference between the groups, according to the education level (Table 6).

Statistically significant difference was obtained regarding the association of stress with **socio-economic** factors in Group 1 vs. Group 2: Job occupation of pregnant women was significantly higher in Group 1, compared to Group 2 (OR = 1.59, p = 0.02, CI = 95%) (Table 7).

Among socio-economic factors, special attention should be paid to overtime work: the Chi-squared test demonstrated, that the percentage of pregnant women working overtime during the whole pregnancy was significantly higher in Group 1, compared to Group 2 (OR = 3.05, p = 0.001, CI = 95%). (Bar Chart 8). The results of the Chi-squared test demonstrated no statistically significant difference between the Group 1 and Group 2 for the following factors: working with the disabled (Chi-squared test = 2.63, p = 0.105); sitting at the computer during the day (Chi-squared test = 2.51, p = 0.113); overnight work (Chi-squared test = 0.22, p = 0.641); lifting heavy objects (Chi-squared test = 2.66, p = 0.103); on feet during the day (Chi-squared test = 0.004, p = 0.945); strenuous mental work Chi-squared test = 0.05, p = 0.816).

The percentage of pregnant women who kept continuing usual work during pregnancy was significantly higher in Group 1, compared to Group 2 (OR = 1.82, p = 0.01, CI95%) (Table 9).

The chi-squared test revealed, that among the environmental factors, the percentage of pregnant women who lived alone or together with a partner, was significantly higher in Group 1, compared to Group 2, where 3+ persons and partner lived together (OR = 1.59, p = 0.03, CI95%) (Table 10).

A statistically significant association was obtained regarding relationship factors, such as having/not having a partner: the percentage of pregnant women with a partner/husband was significantly lower in Group 1, compared to Group 2 (p = 0.01) (Table 11).

Chi-squared test demonstrated statistically significant associations regarding psychological factors, such as: spontaneous and artificial abortions. Both types of abortions were significantly higher in Group 1, compared to Group 2 (Tables 12, 13).

Chi-squared test demonstrated that the marriage registration status of study participants did not differ significantly between the Groups 1 and 2 (Table 14).

The results, obtained from the statistical processing of the data showed, that the sources of the family material provision did not differ significantly between Groups 1 and 2 (Table 15).

Chi-squared test showed no statistically significant difference between the groups regarding the distribution of participants by the flat area per person and also regarding the mean values of flat area per person (Table 16, 17).

The difference between the distribution of participants by the monthly income in the groups was not significant (Table 18).

The chi-squared test showed that the family environment was not significantly different between groups 1 and 2. However, the percentage difference was close to the confidence level (Table 19).

Covid-19 infection case frequency was not significantly different between the groups. However, the difference was close to the confidence level. Chi-squared test = 3.83, p = 0.050 (Bar Chart 20).

The odds ratio (OR) of 1.53 with a 95% confidence interval (CI) of 0.99–2.33 suggests that Group 1 has 53% higher odds of Covid-19 infection compared to Group 2. However, the confidence interval includes 1 (since 0.99 is very close to 1), indicating that this result is not statistically significant at the conventional p=0.05 level. In other words, while the data show a trend where Group 1 may have a higher likelihood of infection than Group 2, this finding could be due to chance because the confidence interval narrowly overlaps with 1. Therefore, further studies with larger sample sizes might be needed to determine whether this observed trend is a true effect.

Free patronage (which means free visits to the gynecologist, financed by the Ministry of Health) of the pregnant women, as well as the data about the regular visits to the gynecologist were not significantly different between groups 1 and 2 (Table 21, 22).

Although some results did not reach statistical significance, these findings remain valuable for the research. They contribute to a more nuanced understanding of the factors influencing stress during pregnancy, suggesting trends and relationships that may warrant further investigation. Non-significant findings also highlight areas where additional research with larger sample sizes or alternative methodologies may provide deeper insights into the complex dynamics of pregnancy-related stress.

## Discussion

This study investigated the associations between various socioeconomic, educational, and cultural factors and the spectrum of stress experienced by pregnant women. The results obtained provide valuable insights into how different stress can impact maternal well-being during pregnancy. In this section, we explore the implications of these findings in the context of existing literature, discuss the potential mechanisms underlying these associations, and consider the areas for future research.

In the study, the participants (N = 398) were divided into two groups based on their stress levels, as confirmed by self-assessment questionnaires and saliva cortisol concentrations. Group 1 consisted of 172 pregnant women with stress, where cortisol levels were randomly measured in a subset of 95 participants. Group 2 included 226 pregnant women without stress, as indicated by their questionnaire responses.

An independent t-test was conducted to compare the mean age between Group 1 and Group 2. The results (t-test = −1.721, p = 0.086) indicated no statistically significant age difference between the two groups (Table 4). Specifically, the mean age of Group 1 participants was 30.4 ± 5.8 years, while Group 2 participants had a mean age of 29.4 ± 5.7 years. This similarity in age distribution between the groups reduces the likelihood that age differences contributed to variations in stress levels observed between the groups. Consequently, it strengthens the validity of our findings by minimizing age as a potential confounding variable.

To explore potential differences in dwelling places between the two groups, a Chi-squared test was conducted. The results (Chi-squared test = 2.91, p = 0.088) indicated that there was no statistically significant difference in dwelling location between groups 1 and 2 (Table 5).

This lack of significant difference suggests that geographic location, at least at the level of capital versus regional residence, may not have played a prominent role in differentiating stress levels in this sample. This finding reduces the likelihood that regional disparities contributed to stress, enabling a clearer focus on other socio-economic, educational, and cultural factors as the potential contributors to the observed stress levels.

Research on stress levels in urban versus rural settings often reveals complex and sometimes non-significant associations between place of residence and stress. For example, some studies indicate that urban living does not inherently increase the likelihood of mental health issues and that rural residents may report similar or even slightly elevated levels of stress in certain contexts. Studies have shown that while stress and mental health challenges are present in both settings, specific factors like social support and community cohesion can vary by location and may influence stress outcomes in nuanced ways (Jeronimo Cortina, Shana Hardin. 2023).

Research conducted in the US has found no strong association between urbanicity and the prevalence of mental health issues after adjusting for other demographic factors. Additionally, studies focusing on family interactions in urban versus rural settings suggest that rural families may experience different stressors that impact family dynamics, but these differences don’t always equate to increased stress levels based on residence alone. In some cases, urban-rural stress levels only differ when considering additional demographic or socio-economic conditions, making it difficult to draw definitive conclusions on stress solely based on dwelling place (Oliver Gruebner, Michael A Rapp. 2017).

To examine the potential impact of education level on stress among study participants, a Chi-squared test was conducted. The results (Chi-squared test = 0.66, p = 0.719) indicated no statistically significant difference in education levels between Group 1 and Group 2 (Table 6). In Group 1, 30.2% had a secondary education, 4.7% had some university education without completing a degree, and 65.1% had completed higher education. Similarly, in Group 2, 31.9% had a secondary education, 6.2% had some university education, and 61.9% had completed higher education.

The lack of a significant difference suggests that educational attainment may not play a central role in differentiating stress levels in this sample. This finding aligns with research indicating that the impact of education on stress may be moderated by other socioeconomic or contextual factors. Thus, the results underscore the need to examine stress through a multi-dimensional lens, considering not only education but also other socioeconomic, cultural, and relational influences.

The analysis revealed a statistically significant difference in socioeconomic stress factors, such as job occupation, between groups 1 and 2 (Table 7). Full-time or part-time employment was significantly more prevalent among the women in Group 1 (59.3%) than in Group 2 (47.8%), with an odds ratio of 1.59 (p = 0.02, CI = 95%). Conversely, a higher percentage of participants in Group 2 were temporarily unemployed or identified as housewives (52.2% vs. 40.7%).

This finding highlights occupational status as a significant factor in stress during pregnancy, with employment demands—such as job security, workload, and work-life balance—likely contributing to higher stress levels. Consistent with previous studies, employment emerges as a key socioeconomic stress factor, especially for pregnant women juggling multiple roles. A 2022 systematic review examined screening and interventions addressing employment as a social risk factor in pregnancy but found that screening tools often overlook job-specific factors like work hours or workplace support (Julia M Goodman, Mina Colon. 2022). Future research should explore these nuances to better inform targeted interventions. Additionally, understanding how employment screening is applied in healthcare settings could enhance strategies to support pregnant women facing employment-related stress.

An examination of socio-economic stress factors related to job demands during pregnancy revealed a significant difference between Group 1 and Group 2 in terms of overtime work (Bar Chart 8). The Chi-squared test indicated that a notably higher percentage of the women in Group 1 (15.7%) reported working overtime throughout their pregnancy compared to Group 2 (5.8%), with an odds ratio of 3.05 (p = 0.001, CI = 95%). This suggests that pregnant women working overtime are over three times more likely to have experienced elevated stress levels than those who do not work overtime.

The impact of overtime work as a stressor during pregnancy may be associated with the increased physical and mental demands it entails, possibly exacerbating fatigue, emotional strain, and overall health risks. While other job-related factors, such as strenuous mental work, physical activity on the job, and overnight shifts, did not show significant associations with stress levels, the findings highlight overtime as a critical area for further investigation and intervention.

A significant difference was observed in the continuation of usual work duties throughout pregnancy between Group 1 and Group 2 (Table 9). In Group 1, 24.4% of pregnant women maintained their regular workload during pregnancy, compared to only 15.0% in Group 2, with an odds ratio of 1.82 (p = 0.01, CI = 95%). The Chi-squared test (Chi-squared test = 5.54, p = 0.019) confirmed that continuing with regular job responsibilities was significantly more common in Group 1, where women also reported higher stress levels.

This result suggests that maintaining a standard workload without adjustments for pregnancy may contribute to elevated stress, potentially due to the physical and psychological demands associated with unmodified job duties. Consistent with findings from similar studies, it appears that the continuation of usual work, without necessary support or accommodations, can be a significant socio-economic stressor in pregnancy. The research, studying the workload and stress during pregnancy (Retno Widowati, Rini Kundaryanti. 2020) demonstrated that workload and working hours are related to the work stress level of pregnant women significantly.

This highlights the importance of flexible workplace policies and the provision of maternity-related adjustments, as these could be vital in reducing stress and supporting maternal well-being. Future research should further explore the types of workplace adjustments that could most effectively mitigate stress for pregnant employees.

The Chi-squared analysis revealed a significant difference in living arrangements between Group 1 and Group 2, particularly regarding the number of people living with the pregnant women (Table 10). The women in Group 1, where higher stress levels were reported, were more likely to have lived alone or with just one other person (34.3%) compared to those in Group 2 (24.8%), with an odds ratio of 1.59 (p = 0.03, CI = 95%). By contrast, the majority of the women in Group 2 (75.2%) lived with three or more people, potentially providing more social support.

This finding suggests that living with fewer individuals may correlate with heightened stress levels, possibly due to limited social interaction or reduced support for daily tasks and emotional needs. Literature on maternal health often underscores the importance of social support in alleviating stress, as increased interaction with family or household members can provide essential emotional and practical support during pregnancy. The results of the systematic review about the women’s social support during pregnancy (Mona Al-Mutawtah, Emma Campbell. 2023) demonstrates a broad variety of emotional support experienced and valued by pregnant women from different sources. Additionally, women expressed satisfaction and dissatisfaction with tangible and intangible support forms.

The present results align with these observations, indicating that the social environment and living arrangements may play a vital role in moderating stress during pregnancy. A significant association was found between relationship status and stress levels in pregnancy, particularly regarding the presence or absence of a partner (Table 11). In Group 1, the proportion of women with a partner or a husband was slightly lower (97.1%) compared to Group 2, where all participants reported having a partner (100.0%). This difference was statistically significant (Chi-squared test = 6.64, p = 0.01).

This result suggests that relationship status may influence stress levels during pregnancy, as the absence of a partner could potentially contribute to increased relational and emotional stress. The absence of a partner’s support may lead to increased stress, as single or divorced individuals may have fewer resources and face additional challenges in managing the physical and emotional demands of pregnancy. This finding aligns with research showing that partner support often correlates with reduced stress and improved maternal health outcomes.

Chi-squared tests showed significant associations between psychological stress and both spontaneous and artificial abortions, with higher rates in Group 1 than in Group 2 (Tables 12 and 13). Spontaneous abortions were reported by 32.0% in Group 1 versus 17.7% in Group 2 (Chi-squared test = 10.93, p = 0.001; OR = 2.19, 95% CI = 1.37–3.49). Artificial abortions were also more common in Group 1 (21.5%) than in Group 2 (11.5%) (Chi-squared test = 7.32, p = 0.007; OR = 2.10, 95% CI = 1.22–3.64). These findings suggest that psychological stress may significantly increase the risk of pregnancy loss, aligning with prior studies linking stress to adverse reproductive outcomes (Miller et al., 2011; Cohn et al., 2019).

The psychological impact of both spontaneous and induced abortions can create a cycle of stress that may exacerbate maternal health issues. Women who experience such events often report feelings of guilt, anxiety, and depression, which can further contribute to stress during subsequent pregnancies (Diana Cuenca 1. 2023).

Analysis of COVID-19 cases showed no statistically significant difference between Group 1 and Group 2, though the result was borderline (Bar Chart 20). In Group 1, 67.4% reported COVID-19 infection 3–6 months before conception, compared to 61.5% in Group 2 (Chi-squared test = 3.83, p = 0.050; OR = 1.53, 95% CI = 0.99–2.33). While this suggests 53% higher odds of infection in Group 1, the result isn’t statistically significant. This trend, however, highlights a possible link between higher stress and infection rates, warranting further research, especially in light of studies indicating potential COVID-19 risks during pregnancy (Kreitel et al., 2021; Allotey et al., 2020) In addition to statistically significant findings, our analysis yielded several results that did not reach statistical significance. Such findings are: The difference in the mean age between groups 1 and 2; the dwelling place of the study participants; education level; marriage registration status; source of the family material provision; flat area (m^2^) per person; monthly Income; family environment. These non-significant outcomes provide valuable insights, as they contribute to a more comprehensive understanding of the research context and suggest areas for future investigation.

## Conclusions

The findings from this study identify key factors contributing to stress during pregnancy, including socio-economic, environmental, relational, and psychological influences. Women living in high-stress environments, facing demanding job conditions throughout pregnancy, experiencing psychological burdens, or lacking spousal support were shown to have heightened stress levels, as evidenced by elevated salivary cortisol concentrations. This significant association between self-reported stress and increased cortisol levels underscores the necessity of holistic prenatal care strategies. Such strategies should not only focus on medical and psychological support but also incorporate social and occupational elements to effectively reduce stress and foster healthier outcomes for both mother and child.

It is important to note that these findings are preliminary and represent intermediary results from an ongoing study. While they provide valuable initial insights into the multifaceted nature of maternal stress, further investigation will enrich our understanding. Completion of this research is expected to yield a more detailed and comprehensive perspective on the impact of maternal stress on infant development.

## Figures and Tables

**Bar Chart 8.**
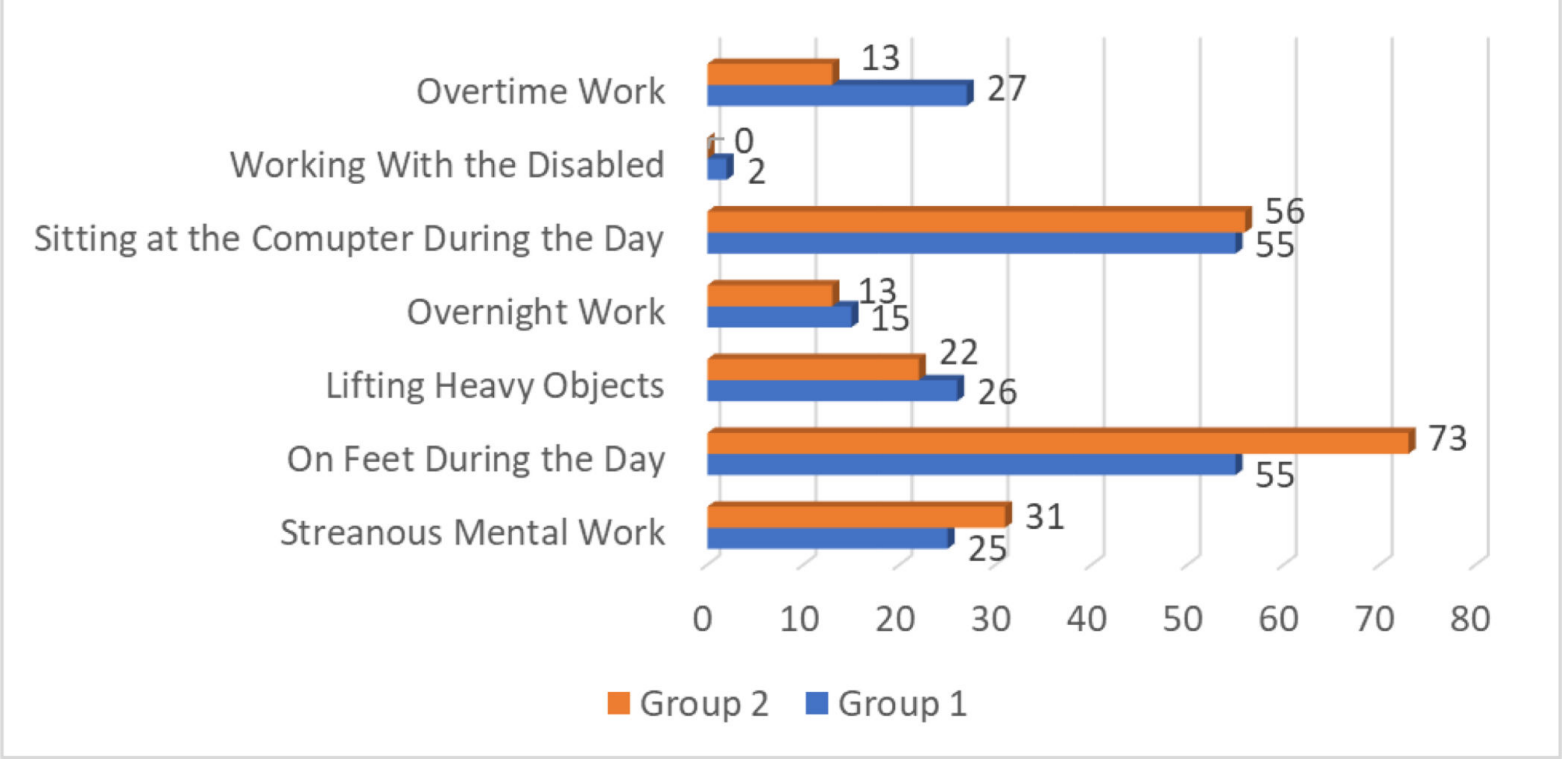


**Bar Chart 20.**
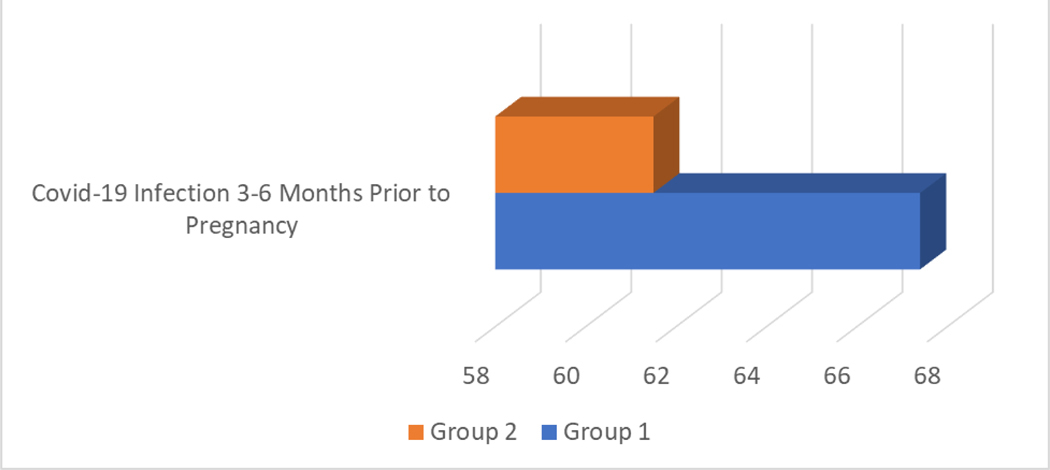


**Table 1.** 

Inclusion criteria	Definition
Gestational age	Pregnant women in their first, second or third trimester were included to focus on different stages of pregnancy
Age	Women aged 18 years and older were eligible to ensure that the participants were legally able to consent and represent adult pregnancy experiences
Residence	Participants had to be residents of the city Tbilisi, Georgia, to maintain a consistent cultural and socio-economic context for the study
Health status	Women with no pre-existing psychological disorders or severe medical conditions (any chronic diseases) that could independently affect stress levels or cortisol concentrations were included to avoid confounding factors
Willingness to Participate	Only women who provided informed consent and agreed to complete the pregnancy questionnaires and to give saliva samples for saliva cortisol concentration measurement - were included
Access to Prenatal Care	Participants were required to be performing regular visits to the gynecologists, receiving regular prenatal care

**Table 2.** 

Exclusion criteria	Definition
Advanced Maternal Age	**Age-Related Risk Factors**: Pregnant women over the age of 40 may be excluded due to the higher likelihood of pregnancy-related complications and stress specific to advanced maternal age, which may not reflect the general population being studied
High-Risk Pregnancy	**Multiple Pregnancies**: Women carrying twins, triplets, or more may be excluded due to the unique stressors and complications associated with multiple gestations, which could confound the research on general pregnancy stress. **Preexisting Complications of Pregnancy**: Women diagnosed with conditions such as preeclampsia, gestational diabetes, placenta previa, or other high-risk pregnancy factors that could affect both physical and emotional stress levels
Chronic or Severe Health Conditions	**Chronic Diseases**: Pregnant women with chronic conditions such as heart disease, uncontrolled diabetes, kidney disease, autoimmune disorders, or other severe medical conditions that could independently influence stress levels and confound the study results. **Mental Health Disorders**: Women with severe untreated psychiatric conditions (e.g., major depression, bipolar disorder, schizophrenia) may be excluded to protect their health and well-being, as well as to avoid confounding the study’s focus on socio-economic, educational, and cultural stress factors.
Medications During Pregnancy	Use of **Psychotropic or Teratogenic Medications**: Women taking medications known to affect mental health, such as antidepressants, antipsychotics, or other psychotropic drugs, may be excluded, as these medications could influence perceived stress levels. **Medications Affecting Pregnancy Outcomes:** Pregnant women on teratogenic medications (e.g., certain anti-seizure drugs, chemotherapy agents) that pose risks to fetal development may also be excluded.
Current Participation in Similar Studies	Pregnant women who are currently enrolled in other research on pregnancy-related stress or interventions (e.g., mindfulness programs or psychological interventions) may be excluded to avoid the overlap of study variables and ensure unbiased data
Significant Language Barriers	**Non-Native Language Speakers without Translation Services**: Women who are not fluent in the language used for the study and cannot access appropriate translation services may be excluded to ensure proper understanding of the consent process and accurate data collection
Fetal Abnormalities	**Known Fetal Genetic Disorders or Malformations**: Women carrying a fetus with known genetic or congenital abnormalities may be excluded, as these conditions can introduce significant stress and may not align with the study’s focus on external socio-economic or educational stress factors
Lack of Informed Consent	**Cognitive or Intellectual Disabilities**: Pregnant women who are unable to provide informed consent due to cognitive impairments may be excluded to ensure ethical research practices and the safety of participants.

**Table 3.** Topics of the questions, included in the pregnancy stress questionnaire

Emotional Well-being	Questions focused on anxiety, depression, and overall emotional state during pregnancy
**Social Support**	**Items that evaluate the availability and quality of support from family, friends, and the community**
**Education**	**Items that evaluated the education level of the participant**
Material Condition	The financial provision of the family
Socio-Economic Factors	Questions that assessed the financial stability, employment status, and access to healthcare services
Cultural Influences	Questions that allowed participants to describe how cultural norms and societal expectations affected their pregnancy experience
Family Circumstances	Relation of the pregnant women with spouse, with the family members

**Table 4.** 

Parameter	Group 1	Group 2
**Age of study participants**	**30.4 ± 5.8**	**29.4 ± 5.7**

**Table 5.** 

Parameter	Group 1	Group 2
**Dwelling place of study participants**		
Tbilisi (Capital of Georgia)	**121 (70.3%)**	**176 (77.9%)**
Other (Regions of Georgia)	**51 (29.7%)**	**50 (22.1%)**

**Table 6.** 

Parameter	Group 1	Group 2
**Education level**		
Secondary	**52 (30.2%)**	**72 (31.9%)**
Admitted at the university, but haven’t graduated	**8 (4.7%)**	**14 (6.2%)**
Higher	**112 (65.1%)**	**140 (61.9%)**

**Table 7.** 

Parameter	Group 1	Group 2
**Occupation**		
Temporarily unemployed / Housewife	**70 (40.7%)**	**118 (52.2%)**
Full-time work / Part-time work	**102 (59.3%)**	**108 (47.8%)**

**Table 9.** 

Parameter	Group 1	Group 2
**Job during pregnancy**		
Not working/On maternity leave	**130 (75.6%)**	**192 (85.0%)**
Keeping the usual workload	**42 (24.4%)**	**34 (15.0%)**

Chi-squared test = 5.54; p = 0.019

Pregnant women, who were on usual work - Group 1 vs. Group 2 – OR = 1.82 (95%CI – 1.10–3.02)

**Table 10.** 

**Parameter**	**Group 1**	**Group 2**
**Number of relatives living together with the pregnant women**		
	**59**	**56**
1–2	**(34.3%)**	**(24.8%)**
	**113**	**170**
3–6+	**(65.7%)**	**(75.2%)**

Chi-squared test = 4.30; p = 0.038

Pregnant women living together 1–2 – Group 1 vs. Group 2 – OR = 1.59 (95%CI – 1.02–2.45)

**Table 11.** 

Parameter	Group 1	Group 2
**Status of marriage**		
Married / Has a partner	**167 (97.1%)**	**226 (100.0%)**
Not Married / Divorced	**5 (2.9%)**	**0 (0.0%)**

Chi-squared test = 6.64; p = 0.010

**Table 12.** 

Parameter	Group 1	Group 2
**Spontaneous abortion**	**55 (32.0%)**	**40 (17.7%)**

Chi-squared test = 10.93; p = 0.001

Spontaneous abortion – Group 1 vs. Group 2 – OR = 2.19 (95%CI – 1.37–3.49)

**Table 13.** 

Parameter	Group 1	Group 2
**Artificial abortion**	**37 (21.5%)**	**26 (11.5%)**

Chi-squared test = 7.32; p = 0.007

Artificial abortion – Group 1 vs. Group 2 – OR = 2.10 (95%CI – 1.22–3.64)

**Table 14.** 

Parameter	Group 1	Group 2
**Marriage registration status**		
Not registered	**43 (25.0%)**	**59 (26.1%)**
Registered in a civil way	**49 (28.5%)**	**64 (28.3%)**
Registered in a religious way	**24 (14.0%)**	**17 (7.5%)**
Registered in a religious and a civil way	**56 (32.6%)**	**85 (37.6%)**

Chi-squared test = 4.67; p = 0.198

**Table 15.** 

Parameter	Group 1	Group 2
**Source of the family material provision**		
Husbands’ parents	**18 (10.5%)**	**19 (8.4%)**
Pregnant women’s parents	**5 (2.9%)**	**5 (2.2%)**
Husband	**73 (42.4%)**	**114 (50.4%)**
Pregnant woman and her husband equally	**68 (39.5%)**	**86 (38.1%)**
Pregnant woman	**8 (4.7%)**	**2 (0.9%)**

Chi-squared test = 7.53; p = 0.110

**Table 16.** 

Parameter	Group 1	Group 2
**Flat area (square meters (m2))**		
<50	**36 (20.9%)**	**34 (15.0%)**
51–100	**62 (36.0%)**	**111 (49.1%)**
101–130	**34 (19.8%)**	**39 (17.3%)**
131–150	**30 (17.4%)**	**35 (15.5%)**
>150	**10 (18.0%)**	**7 (3.1%)**

Chi-squared test = 8.01; p = 0.091

**Table 17.** 

Parameter	Group 1	Group 2
**Mean values of flat area per person (m2)**	**40.2 ± 22.4**	**37.9 ± 17.9**

An independent t-test = −1.138, p = 0.256

**Table 18.** 

Parameter	Group 1	Group 22
**Monthly income (GEL)**		
up to 1000	**21 (12.2%)**	**25 (11.1%)**
1000–3000	**101 (58.7%)**	**146 (64.6%)**
3000+	**50 (29.1%)**	**55 (24.3%)**

Chi-squared test = 1.49; p = 0.476

**Table 19.** 

Parameter	Group 1	Group 2
**Family environment**		
Disputes and tensions between the family members	**17 (9.9%)**	**17 (7.5%)**
Everyone on their own	**4 (2.3%)**	**7 (3.1%)**
Peaceful	**151 (87.8%)**	**202 (89.4%)**

Chi-squared test = 0.88; p = 0.645

**Table 21.** 

Parameter	Group 1	Group 2
**Free patronage of the pregnant women**	**142 (82.6%)**	**182 (80.5%)**

Chi-squared test = 0.26; p = 0.607

Free patronage of the pregnant – Group 1 vs. Group 2 – OR = 1.14 (95%CI – 0.68–1.91)

**Table 22.** 

Parameter	Group 1	Group 2
**Regular visits to the gynecologist**	**171 (99.4%)**	**226 (100.0%)**

Chi-squared test = 1.31; p = 0.252

Regular visits to the gynecologist Group 1 vs. Group 2 – OR = 3.96 (95%CI – 0.16–97.86)

## Data Availability

All data are included in the content of the paper.
